# Guidelines; from foe to friend? Comparative interviews with GPs in Norway and Denmark

**DOI:** 10.1186/1472-6963-10-17

**Published:** 2010-01-16

**Authors:** Benedicte Carlsen, Pia K Kjellberg

**Affiliations:** 1 The Rokkan Centre, University Research, Nygaardsgt 5, 5015 Bergen, Norway; 2 Danish Institute for Health Services Research, Dampfaergevej, Copenhagen, Denmark

## Abstract

**Background:**

GPs follow clinical guidelines to varying degrees across practices, regions and countries, but a review study of GPs' attitudes to guidelines found no systematic variation in attitudes between studies from different countries. However, earlier qualitative studies on this topic are not necessarily comparable. Hence, there is a lack of empirical comparative studies of GP's attitudes to following clinical guidelines. In this study we reproduce a Norwegian focus group study of GPs' general attitudes to national clinical guidelines in Denmark and conduct a comparative analysis of the findings.

**Methods:**

A strategic sample of GP's in Norway (27 GPs) and Denmark (18 GPs) was interviewed about their attitudes to guidelines, and the interviews coded and compared for common themes and differences.

**Results:**

Similarities dominated the comparative material, but the analysis also revealed notable differences in attitudes between Norwegian and the Danish GPs. The most important difference was related to GP's attitudes to clinical guidelines that incorporated economic evaluations. While the Norwegian GPs were sceptical to guidelines that incorporated economic evaluation, the Danish GPs regarded these guidelines as important and legitimate. We suggest that the differences could be explained by the history of guideline development in Norway and Denmark respectively. Whereas government guidelines for rationing services were only newly introduced in Norway, they have been used in Denmark for many years.

**Conclusion:**

Comparative qualitative studies of GPs attitudes to clinical guidelines may reveal cross-national differences relating to the varying histories of guideline development. Further studies are needed to explore this hypothesis.

## Background

It is thoroughly documented that general practitioners (GPs) follow clinical guidelines to varying degrees across practices, regions and countries [1-5] and we also have some knowledge about the GPs' attitudes to guidelines that may explain this variation [6,7]. A systematic review and synthesis of qualitative studies of GPs' attitudes to clinical guidelines found several common themes but no pattern of variation in attitudes between studies from different countries [6]. Reviews have depicted some central themes in GPs' deliberations about following guidelines: Disbelief in evidence, focus on the individual patient (in evidence and practice), reluctance to ration on governments' behalf, the doctor - patient relationship, clinical responsibility (leading to defensive practice or guideline adherence), practicalities (time constraints, competence and equipment), knowledge of and accessibility to guidelines [1,6]. However, while studies of attitudes to guidelines among GPs are numerous in the US, UK, Canada, Australia and the Netherlands, studies from Scandinavia are rare.

An obstacle in comparisons of already existing qualitative interview data from different studies is that the context and content of the interviews vary extensively and there is usually inadequate information about the context to interpret how the findings compare. Still, comparative qualitative interview studies are uncommon.

In this comparative study we started out with a focus group study about GPs' attitudes to national clinical guidelines in Norway and subsequently repeated the study among GPs in Denmark. The Norwegian study confirmed international findings of central concerns about guidelines [8]; e.g. practitioners were concerned about the potential loss of clinical discretion and the holistic approach of general practice, which they feared increased use of clinical guidelines might lead to. However, even though only a fraction of Norwegian clinical guidelines are restrictive, the Norwegian GPs seemed especially sceptical to rationing motives in clinical guidelines issued by the health authorities, and claimed they frequently took the patient's side in opposition to the health authorities.

Norway and Denmark are neighbouring countries with many common characteristics regarding population and welfare and with similar health care systems. However, there are some relevant differences between the organisation of Norwegian and Danish general practice that we expected could be reflected in the GPs' attitudes. In general Norwegian GPs are subject to less government regulation and control than their Danish counterparts, although stricter regulations have been signalled [9]. In many ways Norwegian health authorities have followed the Danish: For example, a patient list capitation system and government issued clinical guidelines and regulations have been common in Danish general practices since the 1950s. A patient list system with partial capitation payment based on the Danish model was introduced in Norway in 2001.

An overview of the total volume of Norwegian guidelines relevant to general practice is lacking, but we know that government guidelines are fewer and of a more recent date in Norway. While some government guidelines are purely based on clinical assessments or giving advice about novel technology, government guidelines are increasingly used as a tool for rationing health care and thus incorporating economic considerations, e.g. since 2002 all new drugs are exposed to a cost-benefit analysis before being accepted for patient reimbursement [10]. Different government led institutions, such as the Directorate for Health and Social Affairs, the Norwegian Medicines Agency, the Knowledge Centre for the Health Services, the Norwegian Board of Health, and the Ministry of Health and Care Services, as well as some independent and private organisations; the Norwegian Medical Association, the Norwegian College of General Practitioners, and pharmaceutical companies, have all produced clinical guidelines for general practice in Norway in recent years. Comparatively fewer guidelines are issued by the government led institutions. The Directorate for Health and Social Affairs has only recently (after these interviews were conducted) set up a web-site - "The Health Library" - where guidelines and educational materials issued from both governmental and other sources since 2000 are accessible http://www.shdir.no/helsebiblioteket.

In Denmark clinical guidelines are primarily issued by The Danish College of General Practitioners (DSAM), The National Board of Health and the Institute for Rational Pharmacotherapy, a partly independent institute under the Danish Medicines Agency. DSAM and The National Board of Health have issued guidelines since the late 1980s and they issue both guidelines regarding innovative technology and restrictive guidelines to secure priority setting. DSAM started developing clinical guidelines to prevent health authorities from monopolising the agenda in guideline development [11]. Their strategy was to issue DSAM guidelines as well as to participate in developing government guidelines of relevance to general practice. Since 1999 DSAM has issued 15 guidelines and participate today in the development of guidelines issued by The National Board of Health, which has issued 8 guidelines since 1999.

The Institute for Rational Pharmacotherapy was established in 1999 "to promote the most rational use of current and future medicinal products with respect to both pharmacological and economical aspects" and is "directed towards both primary and hospital care" [12]. Since 1999 this institute has issued 8 reference programmes and 183 drug assessments.

Hence, it may be argued that Denmark has a more mature primary care system (with regard to guideline implementation and regulation), and we thus expected that GPs' attitudes to national clinical guidelines in the two countries somehow would reflect this development.

## Methods

This is a comparative qualitative study based on focus group interviews with GPs in Norway and Denmark.

### Interviews in Norway

The Norwegian part of the study was conducted in 2007 by the Norwegian author who is a social scientist with long experience with health services research and who had conducted group interviews with GPs on earlier occasions. The Norwegian researcher interviewed six groups of GPs following a semi-structured interview guide. (The main questions appear in Table 1).

**Table 1:** Interview guide

1. A short round about each participant's practice.
2. Are guidelines an important part of your practices?

3. Guidelines you are familiar with and use? Why these?

4. Trust in the evidence and the guidelines?

5. What characterises useful guidelines?

6. To what degree do you feel you should follow guidelines?

7. Arguments for extended use of guidelines?

8. Arguments for restricting the use of guidelines?

9. Do different guidelines give diverging advice?

10. Do you experience challenges in combining guidelines and clinical discretions?

11. To what degree do you see it as an ideal to comply with patients' wishes and share decisions with patients?

12. Do you see guidelines as a challenge or as support in relation to negotiations with patients and your role as gatekeeper?

The sampling was mainly conducted according to convenience and included 27 GPs from the counties of Hordaland (20 GPs) and the capital area of Oslo (7 GPs). The participating GPs were all members of doctors' educational groups and thus familiar with each other. The sample was selected from 11 groups that contacted us after receiving a letter of invitation, which we had sent to a total of 93 group leaders. An overview of the sample compared to the total population of GPs in Norway is presented in Table 2. Both in Norway and Denmark, the great majority of GPs are periodically members of such groups which are obligatory to obtain specialist status and to preserve such status.

**Table 2:** Sample profile compared to all Norwegian and Danish GPs

Variable	Norwegian sample	GPs in Norway*	Danish sample	GPs in Denmark**
Number of GPs	27	3862	18	3.639

Male GPs	67%	68%	56%	62%

Age (mean)	45	47	56,2	53,6

Current list size (mean)	1110	1196	1498	1583

GPs with open list	31%	46%	53%	65%

*Data from the Norwegian Labour and Welfare Organisation and the Norwegian Medical Association: Available at http://www.nav.no/page?id=1073743257 and http://www.legeforeningen.no/index.gan?id=124987 Accessed August, 2007.

**Data from the Danish Medical Association and the Danish Ministry of Health and Prevention: Available at http://www.laeger.dk/portal/page/portal/LAEGERDK/LAEGER_DK and http://www.sum.dk/ Accessed June 2009.

Interviews lasted from one to one and a half hour. The group discussions were recorded and subsequently transcribed by a research assistant. The researcher gave a short introduction before each interview and then acted as facilitator to ensure that all the themes in the interview guide were discussed and that all participants were given opportunities to be heard in the discussions. The participants were asked about what they included in the concept of "clinical guidelines" and were asked to mention and discuss any guidelines they were familiar with. More practical details of the interviews are described elsewhere [8].

### Interviews in Denmark

The Danish part of the study was conducted in 2008 by the Danish author who is a social scientist with a background similar to the Norwegian author. The Danish author interviewed three groups of GPs following the same semi-structured interview guide as did the Norwegian author, and prepared for the interviews by reading the Norwegian interviews and discussing central themes with the Norwegian author.

The sampling was conducted according to convenience and included 18 GPs from the capital area of Copenhagen. The participating GPs were all members of doctors' educational groups and thus familiar with each other. The sample consisted of one group that contacted us after receiving a letter of invitation sent to all group leaders in the capital area, and another two groups that contacted us after receiving a follow up letter of recommendation send by a local GP opinion leader. An overview of the sample compared to the total population of GPs in Denmark is presented in Table 2.

The Danish interviews lasted between one and a half and two hours. The group discussions were recorded and transcribed by a research assistant, and the transcriptions were validated by the Danish author.

### Qualitative analysis

The Norwegian interviews were analysed before we conducted the Danish part of the study. The analysis was conducted by the Norwegian researcher and a Norwegian colleague who is both a researcher and a medical doctor (see acknowledgements). We used thematic content analysis [13] which in short means that the researchers read the transcriptions from the interviews several times, discussed possible core themes and agreed about a system of codes to mark the different themes. The themes partly reflected the themes of the interview guide, but new subjects and ways of categorising them also appeared in the discussions and were thus included in the coding scheme. The emerging themes were first ordered and analysed group by group and finally merged in a theme-based file. The overview of core themes and the interpretation of the results in the Norwegian study are published elsewhere [8].

In the Danish part of the study we aimed at reproducing the Norwegian study as far as possible. The Danish and the Norwegian authors read the transcriptions from the Danish interviews, discussed possible core themes and agreed about at set of codes to mark the different themes. The codes were almost the same as the codes used in the analysis of the Norwegian interviews. The transcriptions of the interviews were coded by the Norwegian author and the coding revised by the Danish author.

Finally we conducted a comparative analysis. Both researchers read both sets of coded interviews and co-operated on a cross-case analysis [14]. What we deemed as typical passages from the interviews were grouped under similar headings for Norwegian and Danish interviews separately and these findings were then brought together in a matrix to obtain a further overview of similarities and differences between the Norwegian and the Danish material. Although similarities dominated the material, we found the differences even more interesting to report. Thus we decided to extract and present citations that represent the most evident cross country difference.

## Results

The general impression from the interviews is that the discussions among Norwegian GPs and Danish GPs are quite similar. For example, their concerns about implementing clinical guidelines in practice, their views on clinical evidence and their attitudes and experiences with the professional role in the doctor - patient relationship are comparable. However, there is also a striking difference, which will be presented in more detail below.

The main views that emerged in both the Norwegian and the Danish interviews were:

• The GPs generally felt unable to keep updated on new treatments and research evidence. Many expressed that they regret this situation. They therefore underlined the need for more short and practical clinical guidelines.

• In addition the participants claimed that they were sometimes sceptical to the evidence clinical guidelines were based on. Still, there was consensus about trust in guidelines developed by the GP organisations.

• The interviews also show that GPs experience a dilemma between standardising practice (guidelines are part of this) and individual treatment of the consulting patient. Patients can also appear demanding, which sometimes lead to negotiations with patients to comply with clinical guidelines.

• The interviews indicate that format, accessibility and implementation strategy influence the use of clinical guidelines.

◦ The GPs claimed that they mainly use guidelines that make work easier, not those who complicate practice.

◦ They found some of the guidelines difficult to access.

◦ Some said they miss one complete source for clinical information and guidelines.

An interesting national difference was evident from an early stage in the analysis. This was a difference of degree, not of substance, but it was striking nonetheless: The Danish group discussions did not reflect the Norwegian concern with economic considerations in guidelines. The Danish GPs demonstrated a much more positive attitude to government guidelines, and they appear more appreciative of government efforts to implement priority setting through guidelines. Although they conveyed some displeasure with obligatory regulations from the The National Board of Health, the Danish GPs appear to have internalised the health authorities' goals for resource allocation (as well as the clinical advantages of standardising practice), accepted the need for rationing and thus see their practice as part of a greater scheme of priority setting. While the Norwegian GPs present themselves as allied with their patients against the authorities' attempt to ration health care, the Danish GPs see a need for rationing. The Danish GPs also claimed they sometimes explain their view to patients when they ration health care to follow government guidelines. Thus the Danish GPs do not convey the same one-sided confidence in and alliance with the patients as we registered in the Norwegian study [8]. Additionally, the Danish GPs' seemed to have less respect for patients' demands than their Norwegian colleagues, even though the Danish GPs also admitted difficulties with rationing healthcare when patients are demanding.

The comparative analysis also suggests that the Norwegian doctors fear a coming development towards more standardisation of practice, less clinical freedom and a strengthening of economic considerations at the expense of clinical concerns in government guidelines. In contrast, the Danish GPs remember that they used to be sceptical to government issued clinical guidelines but acknowledge that their attitudes have changed. Hence they now underline the need to standardise care and secure fair allocation of resources.

In the following two sections we present some characteristic extracts from the Norwegian and Danish focus groups that illustrate the core differences in attitudes to rationing and government guidelines.

### Norwegian interviews

#### Reject rationing. Believe economic considerations should be subsidiary

Dr T: Then you have guidelines that are a bit controlling and that are not necessarily clinically well funded, where it is obviously really a question of saving money, which I find a bit difficult to relate to.

Dr K: So, clinical guidelines that guide you about treatment, as a support in daily practice, they are a plus. But when guidelines come with regulations that are primarily economically motivated, then I feel that the focus is wrong.

Dr S: Because the state medicine's agency has a one-sided focus on saving money. You can't always trust what comes from there.

#### Reject guidelines with hidden rationing motives

Dr M: I am concerned that money is all too important. I am afraid the economic issues will overshadow other important values.

Interviewer: That economic issues are included in the decision without your being aware of it?

Dr M: Yes, that that is what lies beneath it all.

Dr L: Sometimes they [the guidelines, red.] give the impression that they are clinically funded, because they expect such guidelines to be more easily accepted. I tend to feel more in opposition towards economically based guidelines. You become more critical, because if it's only economics, then it's deception.

Dr O: I think it's reasonable from the authorities' side to have guidelines which say we have economic boundaries, which mean we can't do everything we could, but then it should be stated that that is what is happening. And there aren't many people who dare to say that. So I am worried that guidelines come out with an underlying agenda which we don't get to see.

#### Fear that economic considerations may become more important than clinical considerations (development)

Dr G: I feel I focus more and more on the rules of the social security office, really on the finances of the social security; it takes more of my focus, away from focussing on what's best for the patients. Have to kind of remind myself that what's most important is actually what's best for the patients, not the rules the social security have set up to make sure the money doesn't just pour out.

### Danish interviews

#### Used to be sceptical to economic considerations in clinical guidelines, but are positive now (development)

Interviewer: But when it [The National Institute for Rational Pharmacotherapy, red.] was established it was concerned with the health economical rational basis that the medical treatment should not only be the best but also the most cost-effective. Did you notice or remark on this?

Dr S: In the beginning I was a little outraged. Why did they have to go in and decide that?

Dr S: Well, there is an increased respect for it [The National Institute for Rational Pharmacotherapy, red.], at least I think. You check and think: Well, I prescribe these pills; they cost kr. 5 a day whereas the others cost kr. 1, and is that... and the next time a patient comes and I have to initiate a treatment, I might as well prescribe the pills that cost kr. 1.

#### Are of the opinion that rationing is necessary as part of the overall priority setting

Dr R: I too think it's a question of money. It's definitely a question of money. I think You could take that into consideration, definitely, both for the sake of the patient's economy, but also for the sake of everyone's economy. We too pay tax off it. And then you could say that we of course cannot single out in advance who of the patients will start coughing and not, so of course you will try the medicine that's ten times cheaper, that's crystal clear.

Dr Y: Well, I personally have no doubt. You see. Both from the perspective of the collective agreement, the legislative perspective, but also from an ethical, moral and collective state of mind we of course must show regard for the economy.

Dr J: Yes. Not least because, you know, the money is potentially taken from other groups.

Dr Y: Sometimes when I say that I am prescribing the cheapest the patient will say: "It's okay, I have a medicine card", or "I've reached the refund limit". And then I reply: "Well, it still costs what it costs, so it's not you but me and the rest of us, who pays." Now that, I think, is a substantial argument! I think we have a responsibility towards the individual patient's economy, but also towards the economy of the society. I really believe that.

#### Sometimes reject clinical guidelines that they do not find rational

Dr H: How many of you have regarded the heart guideline and had a male patient of approximately 60 years with a total cholesterol of 6.3 and a reasonable blood pressure: 130 over 80. And then you look up in the guideline and see; you can actually make a 10 year projection, and then you see; everyone should be given Simvastatin. Or, maybe 10 percent should not be given it, 90 percent should. Do you comply with it [the guideline, red.]?

Collectively: No.

Dr Y: We don't use it.

## Discussion

As expected when comparing GPs in two relatively similar national health care systems, perceptions of clinical guidelines did not differ much between the GPs in the Norwegian and the Danish studies. This also confirms the meta-study of focus group studies from 2007 which did not find any systematic variation in attitudes to clinical guidelines between countries [6]. The common issues found in both groups of interviews here have all been reported in earlier studies and reviews of GPs' attitudes to guidelines [1,7,15-18].

Nevertheless, we also noted a clear cross-national difference: While the Norwegian GPs in this study appear rather sceptical to economic evaluations incorporated in clinical guidelines, and do not see such clinical guidelines as part of a greater scheme for priority setting, the Danish GPs seem able to combine the need for rationing with their professional identity as holistic generalists. Some of the Danish participants explicitly state that economic evaluations need to be included for the benefit of the public. GPs' concerns about economic vs. clinical standards in guidelines have been noted in a few earlier qualitative studies [17,19].

On the one hand, the similarities are important findings to report because they support the knowledge base, and they are easier to explain than the diverging findings. It is more difficult to explain the dissimilarities, which could for example be results of atypical study samples. However, striking differences merit attention and are interesting because they may lead to new hypotheses about explanations that inspire further studies.

If we for example presume that GPs' attitudes reflect organisational features in the national health care systems, it is relevant to ask whether the difference in attitudes between the two groups of GPs in this study represents stages in an international development of general practice. Whereas Norwegian health care has recently started a process of incorporating economic considerations in government guidelines, Danish GPs are accustomed to applying priority setting. Hence, the Norwegian GPs could be voicing preoccupations about a new situations and how it will form clinical practice, while the Danish GPs have internalised the rationale behind using clinical guidelines for rationing. Thus while the Danish GPs do have to negotiate with patients to follow clinical guidelines and still sometimes end up with acceding to patients' requests, they are not themselves opposed to the motivation implicit in the guidelines.

It follows from this line of argument that as rationing through clinical guidelines is increasingly imposed on GPs, they gradually become more positive to such guidelines as they internalise the ideas behind rationing of health care, or perhaps adapt to a situation they cannot change. The development of primary care in UK would be an interesting case for testing this theory. The British primary care system has in many ways inspired reforms and development of the Danish and Norwegian primary care sectors: The British system is well advanced with a central authoritative guideline source (The National Institute for Health and Clinical Excellence), which produces a steady stream of clinical guidelines. In contrast, Norwegian doctors seem to relate to no more than a dozen clinical guidelines and seem confused about which sources to trust [8,20]. Also with regard to government regulation vs. professional autonomy, the countries differ; British GPs are more closely monitored, and have long experiences with rationing health services because of budget responsibility, especially after the new GP contract of 2004 which implies that GPs' payment partly depends on whether evidence based guidelines are followed. Denmark seems to be in a middle position in this alleged development, both with respect to the range of government guidelines and in terms of regulation of professional discretion. If the theory holds, British GPs should be even more positive to rationing than Danish GPs, but this remains to be tested.

A team of British researchers who have studied how British GPs' manage their clinical practices and professional identity explains the development of British general practice thus [21]: From the late 19^th ^Century the biomedical model based on hospital practice became prevalent and GPs gradually attempted to associate themselves with this model to maintain status. From the 1960s an anti-biomedical wave grew, partly spurred by patient empowerment, and led to the development of a holistic and patient centred approach in general practice. However, in later years, health authorities have increased the evidence based standardisation and regulation of general practice. Interestingly, the empirical results from this qualitative study indicate that even though British GPs currently are practicing according to the biomedical model, the GPs for the time being seem to be upholding their holistic identity i.e. the participants in this study claimed to be practicing holistically in spite of the apparent incongruity with their actual practice. Earlier UK studies suggest the opposite, e.g. in an interview study preceding the 2004 contract Charles-Jones et al found that British GPs were re-adopting a biomedical view on medicine, specialising to increase efficiency and that the holistic approach was being marginalised [22]. Summing up, it is fair to say that, according to current knowledge in the field, there is no unambiguous evidence that British GPs are welcoming rationing and standardisation through clinical guidelines.

Still, scepticism and concerns about future changes in work situation is a well-known psychological phenomenon [23] and fits well with current discourse in the medical literature, e.g. voiced in a comment in *Current Surgery *which recognises the controversies concerning guidelines for cost-control but still argues pro-guidelines as long as guidelines do not become the "demanded standards" of practice enforced by health authorities [24]. However, given that there is a relationship between increased economic considerations and GPs' attitudes to clinical guidelines, it need not be linear. Also, there may be alternative explanations for why different interview studies convey different attitudes among GPs. In depth studies are normally not suitable for generalising because the context in terms of interview questions, terminology, setting, relationship between interviewer and interviewees etc vary between studies. Hence, the differences noted across studies may be related to a range of other factors besides the organisation of the health care systems. Adding to the inevitable contextual differences between interview studies, a review of studies applying focus interviews in primary care research warns that the variation in how the methodology is conducted is tremendous [25].

The great advantage of focus groups is the rich and comprehensive material they yield, which in this case indicates that the differences and similarities noted here are well-founded. Adding to the validity of the study is the researchers' impression that the interviewees seemed relaxed and honest during the group interviews; humour and laughs were frequent, as were enthusiastic discussion. Also, we registered no comments indicating scepticism towards the researchers' or their role in this project.

On the other hand it is difficult to assess the representativeness of the participating GPs, i.e. the study sample. According to the limited data we have, the samples are similar to the average of the GP populations in both countries according to a few background characteristics, but bias according to unobservable factors such as attitudes and personality types remains unknown. Thus we cannot ignore the possibility that the difference in attitudes between the Norwegian and the Danish groups could be the result of peculiarities in the samples. However, the group by group analysis did not indicate marked differences between groups within each country, which indicates that unless there have been very different mechanisms influencing the group leaders' decision to participate in the two countries, the groups at least can be expected to represent the same type of GPs in both countries.

Finally, it is relevant to underline that interviews, whether they are quantitative or qualitative, are fallible as measures of people's behaviour. Hence, attitudes to guidelines are not necessarily a good measure, or even explanation of guideline adherence in practice. Studies frequently report that respondents, both in surveys and interview studies, are notoriously inaccurate when predicting or reporting own behaviour [26,27]. Indeed, a multi-method international study of adherence to clinical guidelines in Norway, the Netherlands and Sweden found that less then 17% of the variation in practice was explained by the attitudes appearing in an included survey [3].

## Conclusions

This study supports earlier reviews suggesting that many, but not all attitudes and dilemmas associated with the use of clinical guidelines in general practice are repeated across national settings. Hence this study may contribute to the cross-national knowledge of how to apply incentives and manage general practice.

To increase our understanding of how GPs react to the development towards increased standardisation of practice and the weight put on cost effectiveness by national authorities, we would like to reproduce this study among British GPs.

## Competing of interests

The authors declare that they have no competing interests.

## Authors' contributions

BC conducted and analysed the Norwegian interviews, BC and PKK planned the Danish interviews. PKK organised and conducted the Danish interviews. BC and PKK analysed the Danish interviews. BC drafted the paper. PKK added information about the Danish part of the study and revised the manuscript. Both authors have read and approved the final manuscript.

## Pre-publication history

The pre-publication history for this paper can be accessed here:

http://www.biomedcentral.com/1472-6963/10/17/prepub
